# Honest Dentistry

**Published:** 1920-09

**Authors:** M. A. Fink

**Affiliations:** New York, N. Y.


### HONEST DENTISTRY

BY M. A. FINK, D.D.S., NEW YORK, N. Y.

While the current professional literature records new inventions, new discoveries, as the result of the efforts of modern research workers, and while discussions and articles about root-canal filling, local conduction and general anesthesia, apicoectomy, tooth replantation, removable and stationary bridge work, pyorrhea alveolaris, and a multitude of allied subjects, fill the pages of the professional magazines, I think it is not amiss to draw at intervals the attention of dentists back to the aim and goal of dentistry. The situation is comparable to that of a person who in examining minutely the details of a splendidly executed monument of Gothic architecture might easily overlook the relation which the detail bears to the whole; so also the dentist who is constantly hearing and reading about all the various topics of his profession may easily fail to keep in mind what really constitutes the ultimate aim and goal of the whole science of dentistry. Another reason for restating this aim from time to time is that new discoveries may have modified, elaborated, or altogether changed our conception of the science.

In my judgment, then, the aim of the science of dentistry is to prevent the necessity for those operations which the dentist is usually called upon to perform, or in other words the prevention of decay and the preservation of the teeth in a healthy and normal condition.

What would bring us nearest to this ideal state? I think I can affirm without the risk of much controversy that in order to attain our object it is imperative:

1. That we maintain the patient's teeth in a perfectly clean and polished condition.

2. That we examine the patient's teeth at frequent intervals.

It is a fact proved by an overabundant amount of clinical evidence that the chances for clean teeth to decay are fully ninety per cent. less than are those for unclean teeth. As a matter of fact, Professor Miller by his classical researches has demonstrated conclusively that decay of teeth is only produced by the action of the zymogenic and saprophytic micro-organisms on nutritive material. The accumulation of nutritive material for bacteria in the mouth indicates an unclean condition which unfortunately exists among millions of people of all classes and of all nations.

If the chances for teeth to decay are eliminated, or at least enormously reduced by keeping them in a clean condition, then it follows as the day follows the night that the best thing a dentist can do is to clean the teeth of his patients and keep them clean and polished. That would be his most glorious achievement. Every dentist will also readily admit that the best artificial restorations, whatever they may be—fillings, crowns, inlays, bridges of all kinds, etc.—are far less efficient than the normal natural organs. Is that not another reason why the preservation of the natural organs is of the utmost importance? If we lose a valuable object, the loss, however great, is never so serious if the lost object can be replaced entirely and exactly. Lost tooth structure can never be so replaced.

If what I have stated is accepted as true, then naturally two questions arise in our minds:

1. Are dentists generally doing prophylactic operations?

2. If not, why not?

Before endeavoring to answer these questions, I wish to say that the conditions I intend to point out not only prevail in the United States, but so far as I know all over Europe and probably all over the world.

Every layman will answer the first of the above-stated questions correctly, and this is in the negative. Extractions, crowns, fillings, bridges, plates, are so much associated with the dentist's activities to the exclusion of everything else that young or old, male or female, will answer something to that effect when asked what is the chief work of a dentist. For many "tooth extractor" or, to be more correct, "tooth puller," seems to be the only correct definition for dentist.

Why the dentist is not doing more preventive work and consequently saving more teeth from destruction requires a somewhat more detailed answer.

In my opinion the principal reasons are:

1. Because the general public has not been taught to appreciate adequately the value of the preservation of the teeth, and when the dentist is visited the ravages of decay have already begun.

2. Because patients are not willing to pay a reasonable fee for prophylactic treatment, which, when properly performed, requires frequently a great amount of time and energy.

3. Because the advertising parlors have given the public wrong impressions and conceptions. Leaving entirely aside the ethical aspect of the question of advertising, the advertisements have been so styled and arranged as to be misleading. This is such a well-known fact to every man in the profession that illustrations would be absolutely superfluous.

4. Because of the charlatans—illegal practitioners.

It is one thing to point out faults, and another and more difficult thing to correct them; but is not the first step to remedy unhealthy conditions a correct diagnosis?

Having enumerated the principal causes for the deplorable conditions generally found in the mouths of the people and the deplorable condition found in the dental profession, I shall now try to suggest the remedies by a mere process of logical deduction:

1. As the evil is so widely spread it is obvious that one alone can not check or overcome it; the intelligent cooperation of all honest-minded men in and outside of the profession is required.

2. Education of the public so that the people realize that the preservation of their teeth is of the utmost importance for their general health; that no part of the human body can be neglected without affecting the efficiency of the entire human mechanism; that the teeth form an integral part of the human economy just as any other organ; that the teeth the same as all other structures of the body are supplied with nerve, blood and lymph vessels and are also subject to the same physiological laws.

3. Induce the people to think. A little common sense will soon enable them to differentiate between the honest man and the charlatan.

I shall make myself a little clearer by illustrating this point.

Every one knows that we do not obtain something for nothing, and anything that is worth obtaining must be paid for. A workman must be paid for his time and labor, a lawyer for his advice, etc., and the more the labor, the better the labor; the more the time spent, the better the advice, the more will have to be paid. That is merely just and equitable.

Every one also knows that dentists, the same as other mortals, can not exist on ideals alone. In order to keep their bodies alive, and also to keep their minds alive, they need carbohydrates, proteins, salts, water, fats, shelter, clothing, etc., and all these commodities can not be paid for with ideals, but must be exchanged for something of a more concrete nature.

It follows, then, that when dentists advertise or make it appear as if they examine, clean or extract teeth free of charge, their services are either not worth paying for, or that they must after all get their fees in some way or other, or that they are philanthropists and so well situated that they can afford to give and are actually giving their time and services for the benefit of mankind merely from altruistic motives. Whether or not the latter is the case can safely be left to the judgment and appreciation of the educated as well as the uneducated public.

The question of the advertising parlors and illegal practitioners is apparently taken care of by the state laws.

But is not a law only effective when it is enforced? Is not a law merely a dead letter when it does not provide punishment in case of contravention? Are the punishments provided for the violation of the laws appertaining to the practice of dentistry adequate? No, and therefore so many think it worth while to take chances.

The question has often been asked if all licensed practitioners are capable and if some illegal practitioners can not perform some operations satisfactorily. A little analysis will clearly show that the public should in any case be warned against men who practice unlawfully.

First of all, a license or diploma is less to be regarded as a distinction than as a safeguard for the general public; it points out to the public those men that are probably qualified to give them the services they seek. The object of a dentist's license is the same as a physician's, a teacher's, or a plumber's—it is to safeguard the public.

Secondly, even if an illegal practitioner could perform some operations satisfactorily, it would be only wise and prudent to shun him. Not only professional skill, but also an honest character, are essential requirements for a practitioner. It is only fair to assume that a man who practices in violation of the law will not show himself more scrupulous in the treatment he accords to his patients.

In summarizing, I wish to say that education of the public and the exposure of the true conditions is the greatest and best remedy for all the evils enumerated. Honest men will profit by it, and the quacks will meet with their deserved fate. The people will preserve their teeth and their health, they will make more visits and fewer payments to dentists, and the dentists will have more patients and fewer artificial restorations to make. Everybody will be benefited. But as long as patients will only pay for restorative and not for preventive work, it is only natural that work will frequently be done in their mouths which is not necessary and often very injurious.—*Dental Cosmos.*

